# Potentiation of the anticancer effect of valproic acid, an antiepileptic agent with histone deacetylase inhibitory activity, by the kinase inhibitor Staurosporine or its clinically relevant analogue UCN-01

**DOI:** 10.1038/sj.bjc.6603132

**Published:** 2006-05-16

**Authors:** W-S Yeow, M F Ziauddin, J B Maxhimer, S Shamimi-Noori, A Baras, A Chua, D S Schrump, D M Nguyen

**Affiliations:** 1 Section of Thoracic Oncology, Surgery Branch, Center for Cancer Research, NCI, NIH, Bethesda, MD, USA

**Keywords:** histone deacetylase inhibitor, valproic acid, UCN-01, Staurosporine, protein kinase C, Parthenolide, NF-*κ*B, apoptosis

## Abstract

Histone deacetylase inhibitors (HDACIs) are novel anticancer agents with potent cytotoxicity against a wide range of malignancies. We have previously demonstrated that either Calphostin C (CC) (a protein kinase C (PKC) inhibitor) or Parthenolide (an NF-*κ*B inhibitor) abrogates HDACI-induced transcriptional activation of NF-*κ*B and p21, which is associated with profound potentiation of HDACI-mediated induction of apoptosis. Valproic acid (VA), a commonly used antiepileptic agent, has recently been shown to be an HDACI. This study was aimed to evaluate the anticancer property of VA in thoracic cancer cells and the development of clinically relevant strategies to enhance VA-mediated induction of apoptosis using kinase inhibitors Staurosporine (STP) or its analogue UCN-01. Treating cultured thoracic cancer cells with VA (0.62–10.0 mM) resulted in significant cell line- and dose-dependent growth inhibition (IC_50_ values: 4.1–6.0 mM) and cell cycle arrest at G1/S checkpoint with profound accumulation of cells at G0/G1 phase but little induction of apoptosis. Valproic acid, being an HDACI, caused significant dose-dependent accumulation of hyperacetylated histones, following 24 h of treatment. Valproic acid-mediated 5–20-fold upregulation of transcriptional activity of NF-*κ*B was substantially (50–90%) suppressed by cotreatment with CC, STP or UCN-01. Whereas minimal death (<20%) was observed in cells treated with either VA (1.0 or 5.0 mM) alone or kinase inhibitors alone, 60–90% of cells underwent apoptosis following exposure to combinations of VA+kinase inhibitors. Kinase inhibitor-mediated suppression of NF-*κ*B transcriptional activity played an important role in sensitising cancer cells to VA as direct inhibition of NF-*κ*B by Parthenolide drastically synergised with VA to induce apoptosis (VA+Parthenolide: 60–90% compared to <20% following single-drug treatments). In conclusion, VA, a well-known antiepileptic drug, has mild growth-inhibitory activity on cultured cancer cells. The weak VA-mediated induction of apoptosis of thoracic cancer cells can be profoundly enhanced either by Parthenolide, a pharmacologic inhibitor of NF-*κ*B, or by UCN-01 a kinase inhibitor that has already undergone phase I clinical development. Combinations of VA with either a PKC inhibitor or an NF-*κ*B inhibitor are promising novel molecularly targeted therapeutics for thoracic cancers.

Chromatin remodelling via histone acetylation by histone acetyltransferases and histone deacetylases (HDACs) is considered as a key element in the dynamic regulation of many genes that play crucial roles in cell growth and differentiation (Kramer *et al*, 2003; Rosato and Grant, 2004). Chromatin structure, in its compact hypoacetylated state, may affect gene expression by blocking the access of transcription factors to their cognate DNA sequences to initiate gene transcription. On the other hand, chromatins with hyperacetylated core histones, due to acetylation-mediated neutralisation of positive charges on lysine residues and disruption of nucleosome structures, assume a relaxed conformation with unfolding of associated DNA that facilitates transcriptional activity (Grunstein, 1997; Richon *et al*, 1998; Struhl, 1998; Wolffe and Kurumizaka, 1998; Sambucetti *et al*, 1999). Histone deacetylases have been shown to be involved in oncogenic transformation by mediating the function of transcription factors in certain forms of haematologic malignancies and possibly in the pathogenesis of solid tumours (Rosato and Grant, 2004). Therefore, identification and clinical development of pharmacologic inhibitors of HDACs for anticancer therapy have attracted a great deal of interest.

Histone deacetylase inhibitors (HDACIs) are structurally diverse chemical compounds that share common biological properties of inducing core histone hyperacetylation leading to gene expression and of mediating potent antitumour effects. Some of HDACIs are either naturally occurring compound like sodium butyrate, a fatty acid metabolite found in high concentration in the lumen of large intestine, or pharmacologic compound such as valproic acid (VA), a commonly prescribed antiepileptic drug whose pharmacokinetics and toxicity profiles are well documented (Brodie and Dichter, 1996; Blaheta and Cinatl, 2002), whereas others are complex chemicals isolated from culture broths of microorganisms (such as Depsipeptide, Apicidin or Trichostin A (TSA)) or synthetic derivatives (for instance MS-275, CI-994). Histone deacetylase inhibitors are subdivided into four fundamental groups: short-chain fatty acids (sodium butyrate, phenylbutyrate, valproic acid), synthetic benzanide derivatives (MS-275, CI-994), cyclic tetrapeptides (Depsipeptide, Trapoxin, Apicidin) and hydroxamic acids (TSA, suberoylanilide hydroxamic acid (SAHA), LAQ8240) (Rosato and Grant, 2004). Histone deacetylase inhibitors induce differentiation, cell cycle arrest and/or apoptosis of cancer cells in culture and *in vivo* animal models (Jaboin *et al*, 2002; Rosato and Grant, 2004; Schrump and Nguyen, 2005). Multiple HDACIs (sodium butyrate, SAHA, Depsipeptide, MS275) have been shown to have anticancer properties in phase I and II clinical trials (Schrump *et al*, 2002; Kelly *et al*, 2003; Piekarz and Bates, 2004; Rosato and Grant, 2004).

The antitumour activity of HDACIs has been attributed to both their ability to inhibit deacetylases (leading to accumulation of hyperacetylated histones and alteration of gene transcription) and their ability to downregulate phenotypic expression of oncoproteins as well as activation of the intrinsic apoptosis-inducing cascade (Ruefli *et al*, 2001; Yu *et al*, 2002; Marks and Jiang, 2005). Histone deacetylase inhibitor-mediated cell cycle arrests or induction of apoptosis are dependent on treatment conditions such as drug concentrations and duration of drug exposure, as well as the intrinsic sensitivity of malignant cells to this class of anticancer agents (Yu *et al*, 2002; Maxhimer *et al*, 2005). Activation of NF-*κ*B transcriptional activity and upregulation of p21 gene expression are frequently observed following HDACI treatments in cancer cells (Burgess *et al*, 2001; Dai *et al*, 2003; Mayo *et al*, 2003). Histone deacetylase inhibitor-mediated activation of NF-*κ*B activity and/or upregulation of p21 gene expression have actually been shown to impede drug-induced apoptosis (Burgess *et al*, 2001; Mayo *et al*, 2003; Nguyen *et al*, 2003, 2004; Rosato *et al*, 2004; Rundall *et al*, 2004; Maxhimer *et al*, 2005). In fact, inhibition of HDACI-induced NF-*κ*B activation by pharmacologic inhibitors of I*κ*B kinase inhibitor (IKK) or by overexpression of kinase-resistant I*κ*B resulted in substantial induction of apoptosis of HDACI-treated cells (Rundall *et al*, 2004; Maxhimer *et al*, 2005). Similarly, depletion of p21 by antisense oligonucleotides sensitises cultured cancer cells to HDACIs (Burgess *et al*, 2001). The molecular pathways that regulate HDACI-mediated NF-*κ*B activation seem to involve PI3K/Akt (Mayo *et al*, 2003) and/or protein kinase C (PKC) signallings (Kim *et al*, 2003; Maxhimer *et al*, 2005). Mayo *et al* (2003) have demonstrated that the ability of HDACIs to increase NF-*κ*B transcriptional activity was not associated with events that stimulated nuclear translocation, but rather related to modulation of transactivation potential of the RelA/p65 subunit of NF-*κ*B via a PI3K/Akt-dependent mechanism. Maxhimer *et al* (2005) have shown that the PKC inhibitor Calphostin C (CC) completely abrogated TSA-mediated NF-*κ*B activation in cultured thoracic cancer cells and this was associated with profound induction of apoptosis following treatment with CC+TSA drug combination. Moreover, Grant and his co-workers (Rosato *et al*, 2002; Rahmani *et al*, 2003a, 2003b) as well as our group (Nguyen *et al*, 2003, 2004) have published multiple reports that described the synergistic interactions between HDACIs and different kinase inhibitors (for instance, Flavopiridol or LY294002) to induce massive apoptosis in leukaemic or thoracic cancer cells *in vitro*. These findings support further preclinical and clinical development of targeted therapeutic strategies that combine inhibition of either NF-*κ*B or PI3K or PKC signallings with HDACIs by using clinically relevant pharmacologies.

Staurosporine (STP) and its analogue UCN-01 were initially developed as PKC inhibitors for cancer therapy (Seynaeve *et al*, 1994). More recent studies have identified these compounds as kinase inhibitors with a broader range of molecular targets *in vivo*, including chk1/2 and PDK1, a kinase that act directly upstream of Akt and MAP/extracellular signal-regulated kinase (ERK) kinase (MEK) (Sato *et al*, 2002). Exposure of cultured cancer cells to UCN-01 (250–1000 nM) resulted in complete abrogation of phosphorylation at Ser473 and Thr308 residues of Akt, both of which are essential for full activation of Akt kinase activity (Amornphimoltham *et al*, 2004; Kondapaka *et al*, 2004). Clinical trials of UCN-01 in patients with refractory neoplasms, as monotherapy or in combination with cytotoxic chemotherapeutics, have been reported (Sausville *et al*, 2001). Continuous 72-h infusion of UCN-01 resulted in ‘free’ (after ultracentrifugation) plasma drug concentrations of 200–600 nM and tissue drug concentration (via measurement of salivary UCN-01 concentrations as surrogates) of 110 nM (Sausville *et al*, 2001). In view of the facts that STP and its clinically relevant analogue UCN-01 inhibit multiple kinases known to influence the cytotoxic effect of HDACIs in cancer cells, we hypothesised that STP or UCN-01 would synergise with HDACIs to promote profound induction of apoptosis. To this end, we sought to investigate the cytotoxicity of the UCN-01+VA combination in cultured thoracic cancer cells *in vitro*. These agents have been used in human for different indications, the former as a phase I anticancer agent and the latter as an established antiepileptic drug that was recently demonstrated to have anticancer activity *in vitro* and *in vivo* animal model (Gottlicher *et al*, 2001). This combination has the potential to be developed into a clinically applicable novel targeted molecular therapeutics.

## MATERIALS AND METHODS

### Cells and reagents

The oesophageal cancer (EsC) cells TE2, TE12; the non-small-cell lung cancer (NSCLC) cells H322, H460 and the malignant pleural mesothelioma (MPM) cells H513, H211 were maintained in RPMI-1640 culture medium supplemented with fetal calf serum (10% vol vol^−1^), streptomycin (100 *μ*g ml^−1^), penicillin (100 U ml^−1^) and glutamine (2 mM). Trichostatin A (Sigma, St Louis, MO, USA), Calphostin C, STP, (Calbiochem, La Jolla, CA, USA) and the NF-*κ*B inhibitor Parthenolide (Alexis, San Diego, CA, USA) were dissolved in dimethyl sulphoxide and stored at −20°C. Valproic acid (Alexis) was dissolved in distilled water to make 500 mM stock and stored in 4°C. UCN-01 was provided by the Developmental Therapeutic Programme of the National Cancer Institute, NIH, Bethesda, MD, USA.

### Determination of apoptosis

Cancer cells seeded at 3 × 10^5^ cells per well in six-well plates were treated either with VA alone (1.0. or 5.0 mM) or in combination with CC (1.0–2.0 *μ*M), UCN-01 (250–500 nM), STP (200 nM) or Parthenolide (20 *μ*M). The second drug was added into the VA-treated cells 12 h after the onset of VA treatment as 10 × stock solutions with VA remained in the cultured medium. Treated cells were harvested at 48 h after the onset of VA treatment, fixed in 1% paraformaldehyde and 70% ethanol, and assayed for apoptosis by using the terminal deoxynucleotidyltransferase-mediated dUTP nick-end labelling (TUNEL)-based ApoBrdU assay (BD Pharmingen, Torrance, CA, USA), as per the protocol provided by the manufacturer.

### NF-*κ*B transcriptional activity

The transcriptional activity of NF-*κ*B was evaluated by transient transfection of NF-*κ*B-luciferase reporter plasmid (Invitrogen, Carlsbad, CA, USA) into cells before drug treatments. Luciferase activity of cell lysates is directly correlated with the transcriptional activity of NF-*κ*B. Cells were plated onto 24-well plates at a density of 8 × 10^4^ cells well^−1^. For transcription experiments, cells were transfected with 200 ng of NF-*κ*B-Luc plasmid per well with Fugene (Promega, Madison, WI, USA). At 24 h after transfection, cells were treated with different experimental conditions as outlined in the respective figure legends. The transcriptional activity of NF-*κ*B was quantified by measuring the luciferase activity from cell lysates with Luciferase Assay System (Promega, Madison, WI, USA) and the Lumat LB 9507 lucinometer (EG&G Berthold, Gaithersburg, MD, USA). The NF-*κ*B activity was normalised with the total cellular protein in cell lysates determined by BCA protein assay (Pierce Biotechnology, Rockford, IL, USA) and expressed as fold of the NF-*κ*B activity of untreated control cells.

### Western blotting

Cultured NSCLC and EsC cells were treated with different experimental conditions as described in the figure legends. Whole-cell extracts were prepared in cell lysis buffer (Cell Signaling Technologies, Beverly, MA, USA) supplemented with 1 mM PMSF (Sigma, St Louis, MO, USA). In total, 50 *μ*g of cell extracts were separated on a gradient 4–20% SDS–PAGE and transferred onto nitrocellulose membranes. The membranes were blocked in 5% Blotto before immunoblotting with antibodies targeting apoptotic proteins Bax, Bak, Bcl2, BclXL (Cell Signaling Technology, Beverly, MA, USA at 1 : 1000 dilution) and cIAP1 (R&D, Minneapolis, MN, USA, 1 : 2000 dilution). Nitrocellolose membranes were also immunoblotted with antibodies targeting phospho-Adducin (Upstate, Waltham, MA, USA, 1 : 1000 dilution with 3% Blotto), Akt and phospho-Akt Ser473 (Cell Signaling, 1 : 500 dilution with 5% BSA), p42/p44 and phospho-p42/p44 (Cell Signaling, 1 : 1000 dilution with 5% BSA) and acetylated-Histones 3 and 4 (Upstate, 1 : 1000 dilution with 3% Blotto). The primary antibodies were detected by HRP-conjugated anti-mouse and anti-rabbit secondary antibodies and detected with West Dura chemiluminescence (Pierce Biotechnology, Rockford, IL, USA).

### Data analysis

Supra-additive apoptosis is defined as the apoptosis induced by the drug combinations that is, by statistical analysis, significantly greater than the algebraic sum of apoptosis induced by individual drug treatment. Data are expressed as means±standard error of the means (s.e.m.) of at least three independent experiments that yielded similar results. Statistical analysis was performed with the GraphPad InStat software (GraphPad, San Diego, CA, USA). Statistical analysis was performed using analysis of variance (ANOVA) and pairwise comparison using Bonferroni test or by two-tailed Student's *t*-test, with *P*<0.05 indicating statistical significance.

## RESULTS

### Staurosporine or UCN-01 suppresses TSA-mediated NF-*κ*B activation and enhances TSA-induced apoptosis

**Figure 1:**
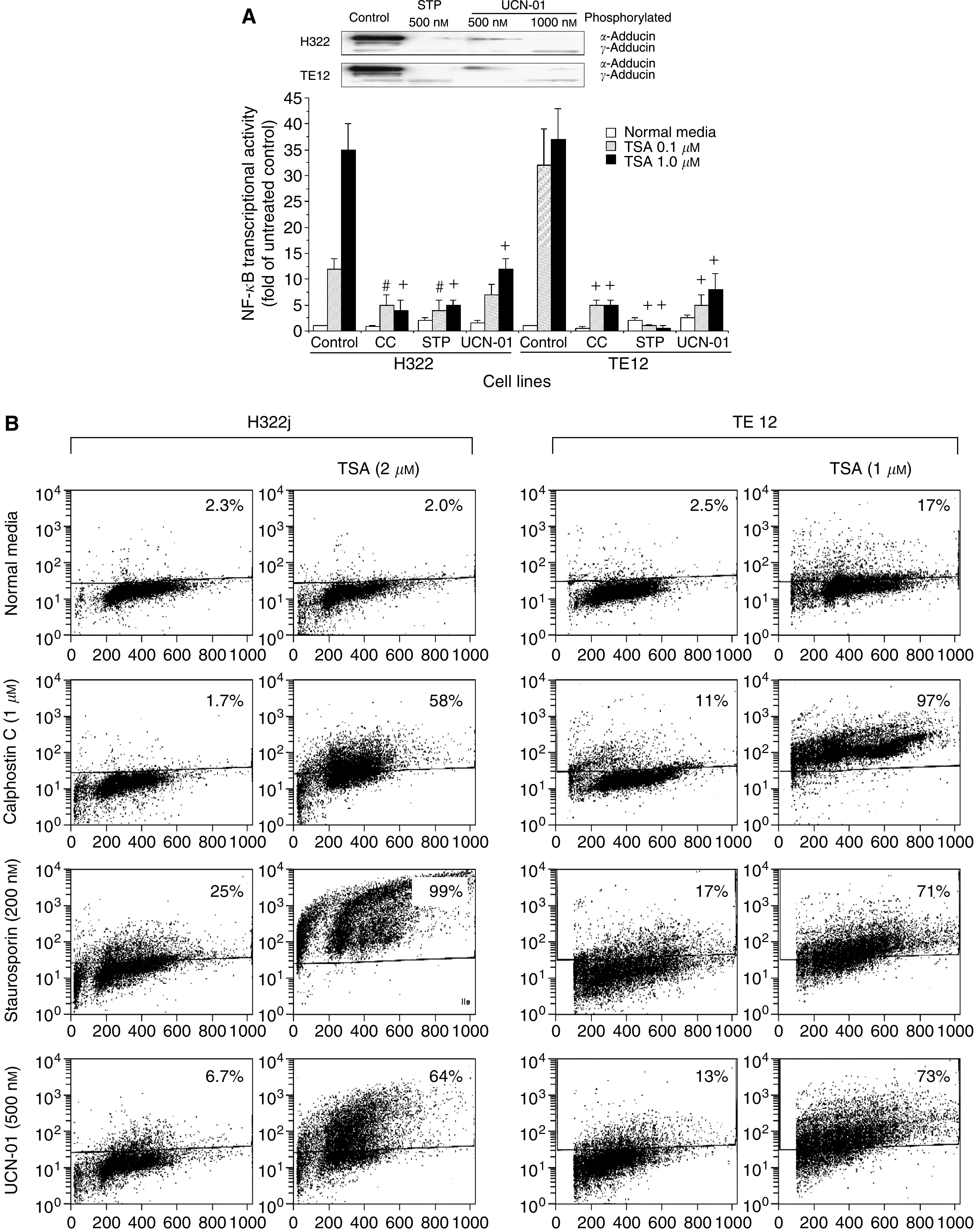
(**A**) Suppression of TSA-mediated upregulation of NF-*κ*B transcriptional activity by Calphostin C, Staurosporine and its analogue UCN-01 in NSCLC cell H322 and EsC cell TE12. Cells were transiently transfected with NF-*κ*B-Luc plasmid as described in Materials and Methods and then subsequently treated with TSA (0.1 and 1.0 *μ*M) with or without concurrent Calphostin C (CC – 2 *μ*M), Staurosporine (STP – 200 nM) or UCN-01 (500 nM). Staurosporine and UCN-01 exerted a potent PKC-inhibitory activity as evidenced by the complete depletion of p-adducin following 12 h of exposure to the respective drugs. Luciferase activity assayed 24 h after drug treatments were normalised for proteins of cell lysates and expressed as fold of activity of untreated control cells. Data are expressed as mean±s.e.m. of three independent experiments (#*P*<0.05–0.01 and +*P*<0.001 *vs* controls by ANOVA and pair-wise comparison by Bonferroni test). (**B**) Profound induction of apoptosis in H322 and TE12 cells treated with TSA+CC, TSA+STP and TSA+UCN-01 combinations. Cells were exposed to TSA (1 or 2 *μ*M) for 12 h, to CC (1 *μ*M) or STP (200 nM) or UCN-01 (500 nM) continuously for 36 h or TSA followed by other drugs. Cells were then harvested 48 h after the onset of treatment to assay for apoptosis. Representative data of three independent experiments that yielded similar results are shown here.

**Figure 2:**
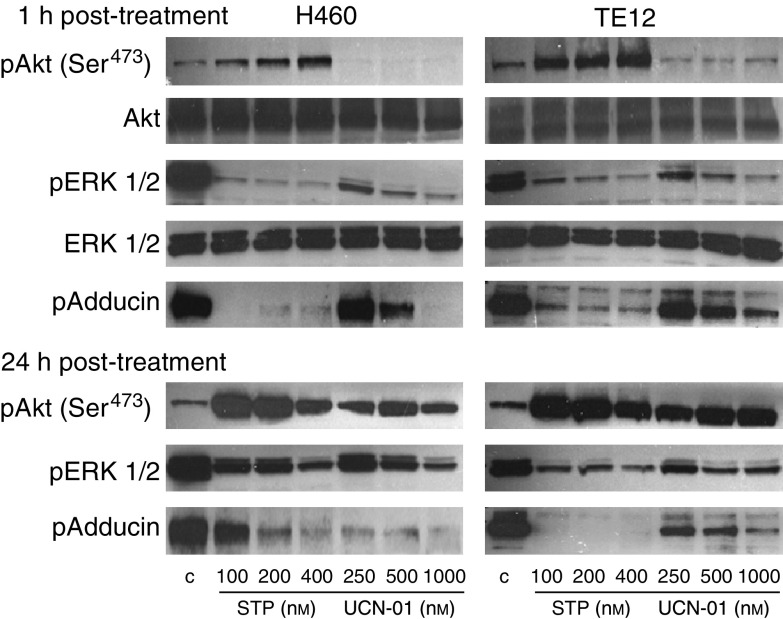
Time-course (1 and 24 h) and dose-dependent STP (50, 100, 200 nM)- or UCN-01 (250, 500, 1000 nM)-mediated modulation of the PKC activity and of phosphorylated Akt, ERK1/2 levels in H460 and TE12 cells. *β*-Actin of cell lysates was blotted to indicate equal loadings of proteins. Profound depletion of pERK1/2, p-adducin and pAkt (in early time points) following UCN-01 exposure. Staurosporine effect on these molecular targets was similar to the effect of UCN-01, except that it stimulated (instead of inhibited) Akt phosphorylation.

As we have previously demonstrated that pharmacologic inhibition of PKC activity by using CC resulted in abrogation of TSA-mediated NF-*κ*B activation and enhancement of apoptosis in cells treated with the TSA+CC combination (Maxhimer *et al*, 2005), we sought to determine if STP or UCN-01, via being PKC inhibitors as indicated by their ability to markedly reduce the expression of phosphorylated *α*/*γ* adducin (p-adducin) (Figure 1A), could duplicate the effect of CC in suppressing NF-*κ*B activation and enhancing the cytotoxic effect of TSA in cultured thoracic cancer cells. Staurosporine (200 nM) or UCN-01 (500 nM) significantly inhibited NF-*κ*B transcriptional activation in H322 and TE12 cells that were treated with TSA, quite similar to the effect of the PKC inhibitor CC (Figure 1A). Whereas less than 20% of cultured thoracic cancer cells exposed to either CC, STP or UCN-01 alone or TSA alone were apoptotic, 55% to more than 90% of cells treated with the respective drug combinations had undergone apoptosis (Figure 1B). However, the supra-additive induction of apoptosis by UCN-01 or STP in combination with TSA may also be attributable to their function as negative modulators of Akt and MEK/ERK1/2 activation via inhibition of PDK1 (Sato *et al*, 2002), in addition to being PKC inhibitors. To further investigate the effect of STP or UCN-01 on MAPK ERK1/2 or Akt activation in cultured thoracic cancer cells, dose–response and time-course studies were performed on cells treated with STP (100–400 nM) or UCN-01 (250–1000 nM) and harvested at 1 or 24 h after the onset of drug exposure (Figure 2). UCN-01 treatment resulted in a significant dose-dependent reduction of pAkt as early as 1 h after drug exposure. At 24 h, higher phosphorylated Akt levels were observed in UCN-01-treated cells than in untreated controls. Staurosporine, on the other hand, mediated a dose-dependent activation of Akt throughout the whole time-course experiment. Dose-dependent suppression of ERK1/2 activation, however, was observed in both STP- and UCN-01-treated cells at both time points. As expected, PKC activity in cultured thoracic cancer cells, as indicated by p-adducin levels, was completely abrogated by either STP or UCN-01, with STP mediating a very rapid onset of PKC inhibition and UCN-01 mediating a slower onset of PKC inhibition.

### The growth-inhibitory effect of VA in cultured thoracic cancer cells

**Figure 3:**
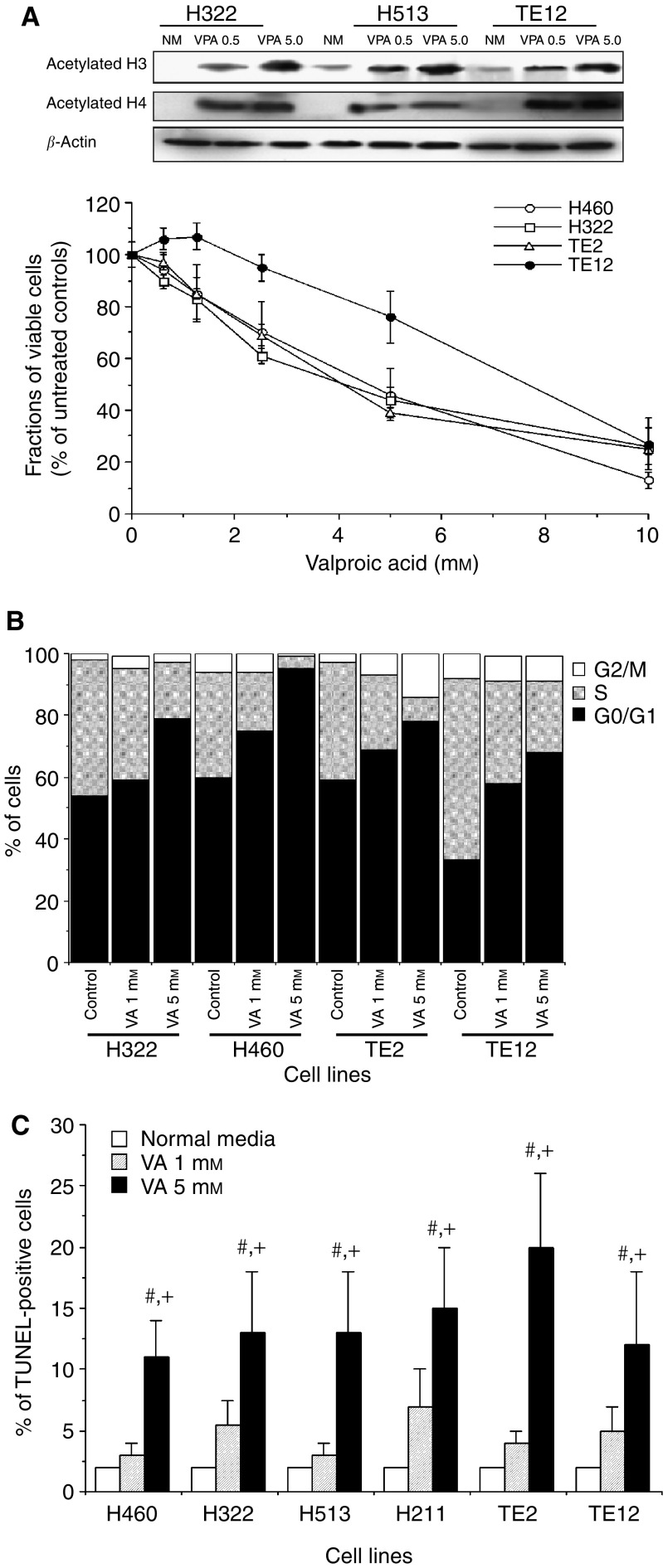
(**A**) The HDAC-inhibitory activity of VA in H460, TE12 and H513 cells. Cells were treated with VA (0.5, 1.0 and 5.0 mM) for 12 h and hyperacetylated H3 and H4 histone proteins were determined by Western blot analysis. Mild growth-inhibitory effect of VA on cultured thoracic cancer cells. Cells, seeded in 96-well microtiter plates, were continuously exposed to VA (0.62–10.0 mM) for 96 h. Cell viability was measured by MTT (4,5-dimethylthiazo-2-yl)-2,5-diphenyl tetrazolium bromide) and cell viability was expressed as percentages of untreated control cells. Data are expressed as mean±s.e.m. of three independent experiments. (**B**) Cell cycle arrest at G1/S checkpoint with accumulation of cells at G0/G1, little induction of apoptosis only at high concentrations of VA (5 mM) for 48 h. (**C**) Mild but statistically significant induction of apoptosis of cultured thoracic cancer cells was only observed following exposure to high concentration of VA (5.0 mM; # or +*P*<0.05–0.01 *vs* control cells or VA (1mM)-treated cells, respectively, by ANOVA and pairwise comparison by Bonferroni test). Cells were continuously treated with VA at either 1.0 or 5.0 mM for 48 h and harvested for quantitation of apoptosis by the TUNEL-based ApoBrdU assay and flow cytometry. Data are expressed as mean±s.e.m. of three independent experiments.

Subsequent experiments were devoted to evaluating the antitumour effect of VA in cultured thoracic cancer cells. Valproic acid has been shown to have HDACI activity and to mediate differentiation and/or apoptosis in different carcinoma cell lines *in vitro* and *in vivo* (Gottlicher *et al*, 2001; Phiel *et al*, 2001; Kramer *et al*, 2003; Gurvich *et al*, 2004). Valproic acid also exhibited HDACI activity in cultured thoracic cancer cells. Treating representative NSCLC cells H460, EsC cells TE12 and MPM cells H513 with VA resulted in a dose-dependent accumulation of hyperacetylated H3 and H4 histones (Figure 3A). Valproic acid mediated a mild reduction of cell proliferation, the magnitude of which was cell line-dependent with the VA IC_50_ values ranging from 4.5 to 8.0 mM (Figure 3A). The effect of VA on the progression of cancer cells through the cell cycle was determined by PI staining and flow cytometry. Treating cultured thoracic cancer cells with VA (1.0 or 5.0 mM) for 48 h resulted in accumulation of cells in G0/G1 phase and a concomitant reduction of cell in S and G2/M phases, indicating that VA mediated cell cycle arrest at the G1/S checkpoint (Figure 3B). Similar to previous reports (Gottlicher *et al*, 2001; Kramer *et al*, 2003), VA was a weak inducer of apoptosis in thoracic cancer cell lines as less than 25% of cells were apoptotic following 48 h of continuous exposure to high concentration of VA at 5.0 mM (Figure 3C). By itself, VA exerted a very mild growth-inhibitory effect, yet we postulated that the cytotoxic effect of VA, being an HDACI, could be enhanced by the PKC inhibitor CC or the broader spectrum kinase inhibitor STP or UCN-01. These kinase inhibitors negatively regulate PKC and ERK1/2 activation, both of which have been shown to potentiate the cytotoxic effect of HDACIs (Rahmani *et al*, 2003b; Maxhimer *et al*, 2005).

### Profound enhancement of apoptosis induction by combining VA with kinase inhibitors

**Figure 4:**
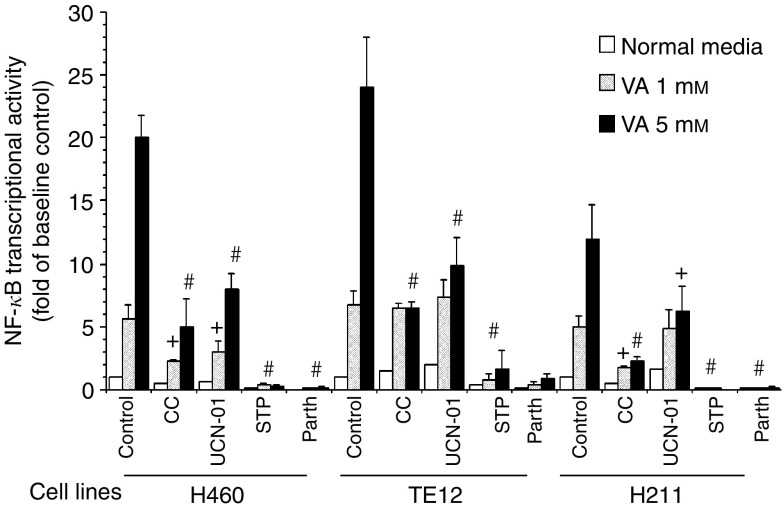
Suppression of VA-mediated activation of NF-*κ*B by Calphostin C (CC), Staurosporine (STP), UCN-01 or Parthenolide in H460, TE12 and H211 cells. Cells were transiently transfected with NF-*κ*B-luciferase reporter plasmid and concurrently treated with VA (1 or 5 mM) in combination with CC (2 *μ*M), STP (200 nM), UCN-01 (500 nM) or Parthenolide (20 *μ*M). Cells were harvested 24 h after the onset of drug treatment and assayed for luciferase activity. Data are presented as fold increase of luciferase activity, normalised for cellular proteins, from baseline activity of untreated controls (means±s.e.m. of three independent experiments; ^+^*P*<0.05–0.01 and ^#^*P*<0.001 *vs* controls by ANOVA and pairwise comparison by Bonferroni test).

**Figure 5:**
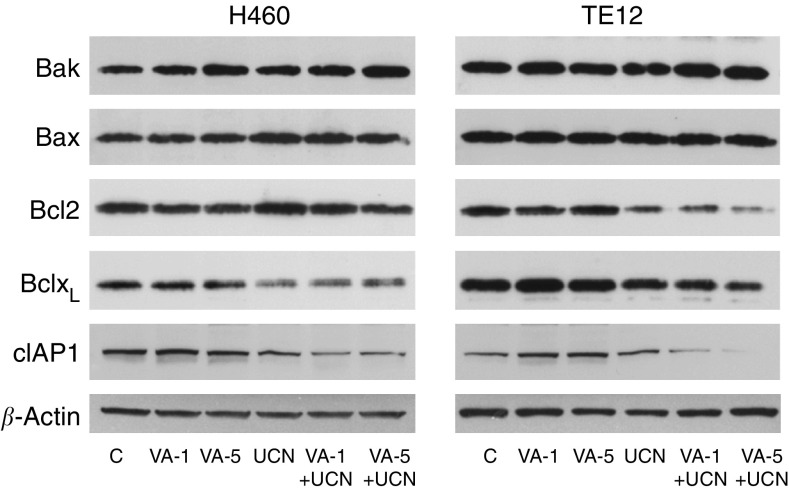
Reduction of Bcl2, BclXL, cIAP1 levels without alteration of the expression of Bak or Bax in TE12 or H460 cells treated with VA (1.0 or 5.0 mM) and UCN-01 (500 nM) concurrent combinations. Representative data of two independent experiments with similar results are shown here.

**Figure 6:**
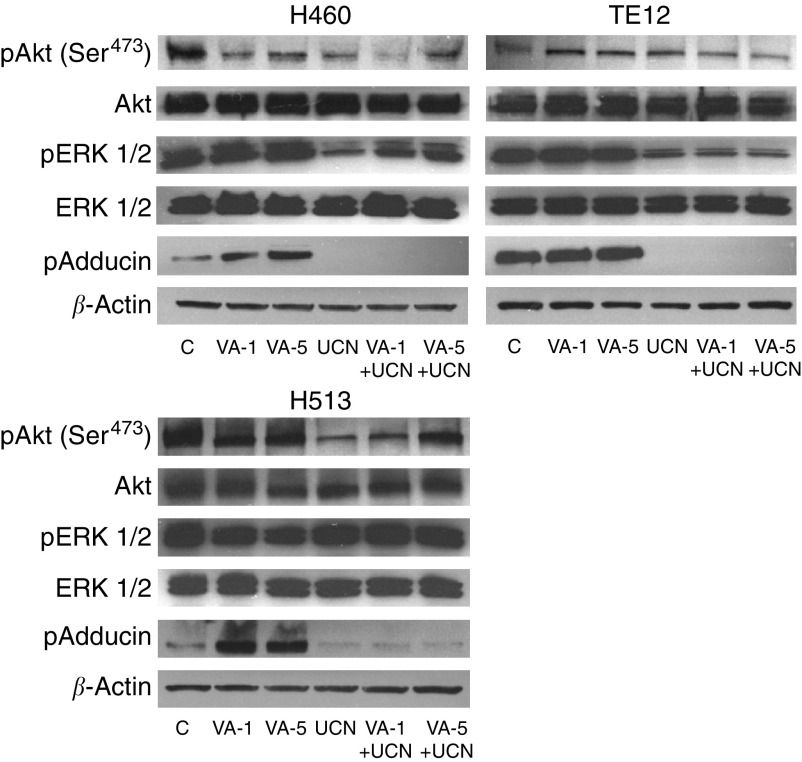
Suppression of pERK1/2, pAkt and p-adducin levels in VA (1.0 or 5.0 mM)-treated H460, TE12 and H513 cells by UCN-01 (500 nM). Representative data of two independent experiments with similar results are shown here.

We first determined if VA, as an HDACI, would induce activation of NF-*κ*B transcriptional activity that could be suppressed by kinase inhibitor CC, STP or UCN-01 as was previously demonstrated with TSA. Treating NSCLC cells H460, EsC cells TE12 or the MPM cells H211 with VA (1.0 or 5.0 mM) for 24 h resulted in a cell line- and dose-dependent 5–20-fold increase of NF-*κ*B transcriptional activity (Figure 4). Such robust upregulation of NF-*κ*B activity was significantly suppressed by CC (2 *μ*M), STP (200 nM) or UCN-01 (500 nM) when assayed 24 h after exposure to the respective drug combinations (Figure 4). Staurosporine was most effective among the kinase inhibitors at inhibiting VA-mediated NF-*κ*B activation. UCN-01, on the other hand, in best experimental conditions mediated 50% (two-fold) to 70% (three-fold) reduction of VA-mediated NF-*κ*B activation. Moreover, there was significant reduction in the levels of the antiapoptotic proteins cIAP1, BclXL and Bcl2 without discernible alterations in the levels of proapoptotic proteins Bax, Bak in H460 or TE12 cells treated with the VA+UCN-01 combinations (Figure 5). The lack of alteration of Bax and Bak, taken together with reduction of the levels of antiapoptotic proteins in cancer cells exposed to the VA+UCN-01 combination, would favour a more proapoptotic milieu within treated cells. Moreover, the effect of UCN-01 on ERK1/2, Akt and PKC activity in VA-treated cells was evaluated by Western blotting for levels of pERK1/2, pAkt and p-adducin (Figure 6). Valproic acid mediated a dose-dependent upregulation of PKC kinase activity as indicated by increased p-adducin levels in H460 and H513 cells, whereas there was no discernable alteration of the already high basal p-adducin level in TE12 cells and these were completely abrogated by UCN-01. Similarly, UCN-01 significantly inhibited ERK1/2 activation in VA-treated H460 and TE12 cells but not H513 cells. On the other hand, UCN-01 mediated a clear depletion of pAkt in H513, H460 and TE12 cells. The VA-induced accumulation of hyperacetylated histone H4 in H460, H513 and TE12 cells was not altered by UCN-01 (data not shown), implying that the synergistic interaction between STP or UCN-01 and VA to mediate suppression of NF-*κ*B transcriptional activity and induction of apoptosis was independent of the HDAC-inhibitory activity of VA (Figure 6).

**Figure 7:**
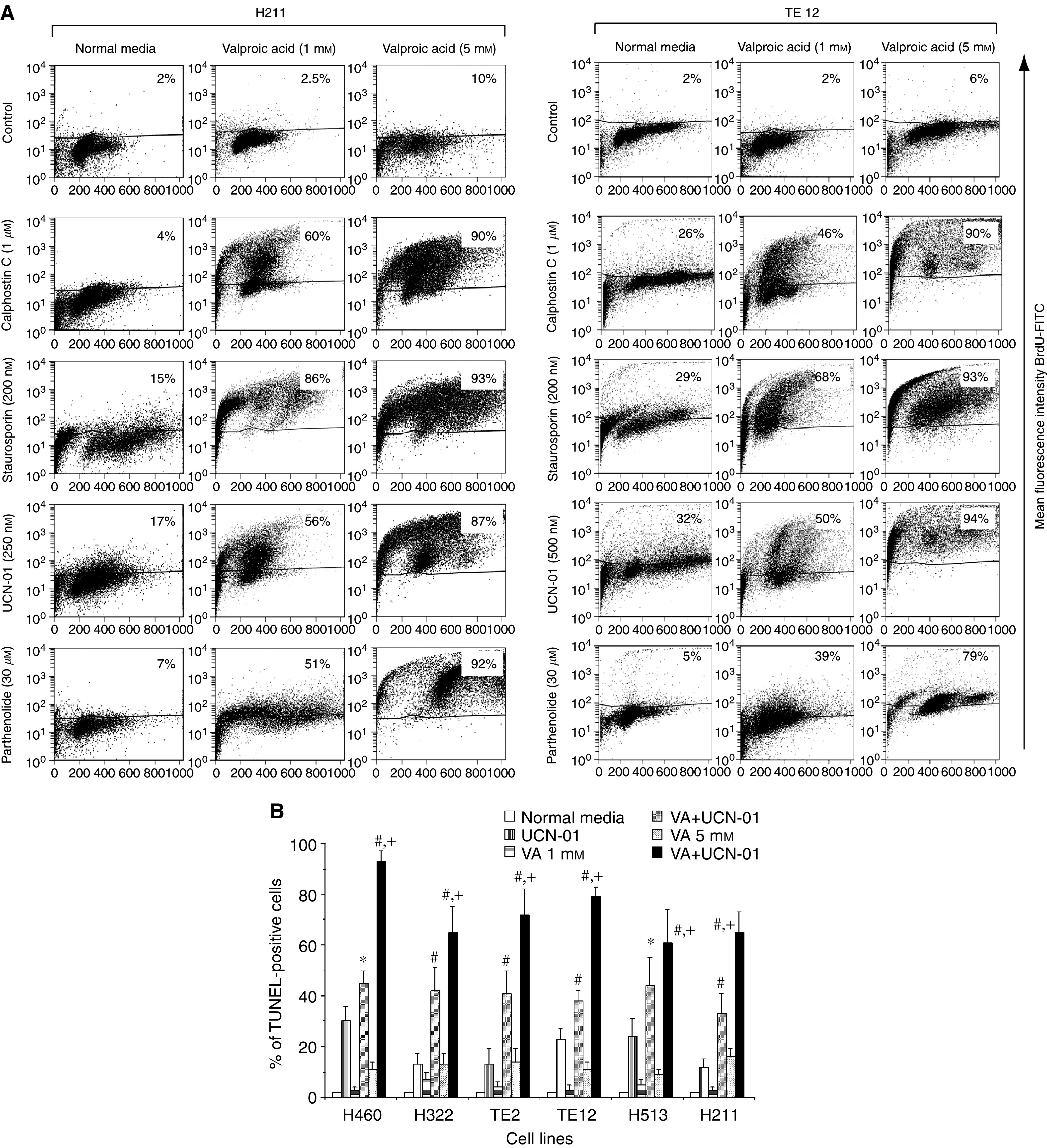
(**A**) Profound enhancement of apoptosis by the combinations of VA (1.0 or 5.0 mM) with CC, STP, UCN-01 or Parthenolide in the representative cultured thoracic cancer cells TE12 or H211. Apoptosis was quantified by TUNEL-based ApoBrdU assay and flow cytometry. (**B**) Summary of the VA dose-dependent additive or supra-additive induction of apoptosis in the panel of six cultured thoracic cancer cells treated with VA and UCN-01 (500 nM) combinations. Additive enhancement of apoptosis was observed in H460 and H513 cells treated with VA (1.0 mM+UCN-01; ^*^P>0.05 combination effects *vs* sum of individual drug effects) and supra-additive enhancement of apoptosis was observed in other cell lines and combinations, especially at the clinically relevant concentration of VA of 1.0 mM (# *P*<0.05–0.001 combination effects *vs* sum of individual drug effects). The magnitude of apoptosis induced by VA+UCN-01 was clearly dependent on VA concentrations (^+^*P*<0.05–0.001 VA(1.0)+UCN-01 *vs* VA(5 mM)+UCN-01). Data are expressed as mean±s.e.m. of three independent experiments.

**Figure 8:**
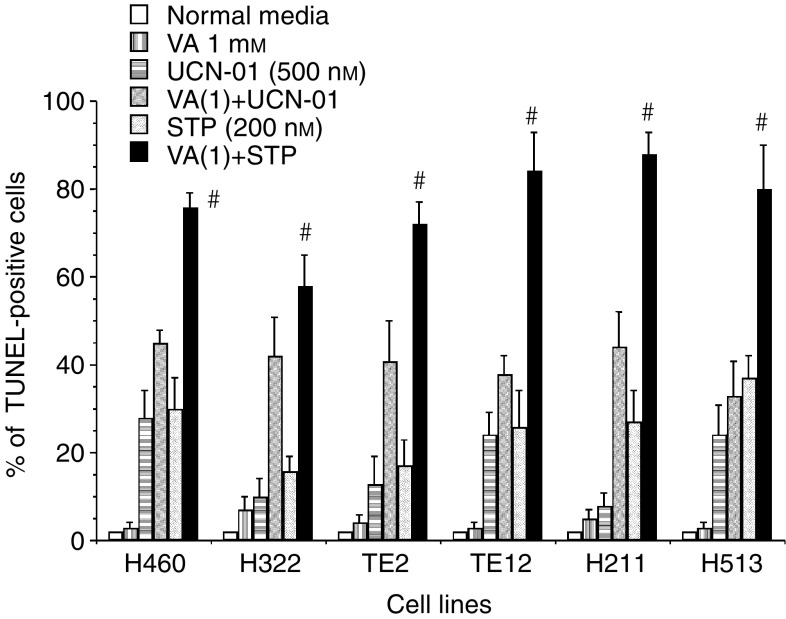
Staurosporine (200 nM) is more potent than UCN-01 (500 nM) in mediating supra-additive enhancement of apoptosis in combination with low concentration of VA of 1.0 mM (^#^*P*=0.0022–0.0001 VA+STP *vs* VA+UCN-01). Data are expressed as mean±s.e.m. of three independent experiments.

**Figure 9:**
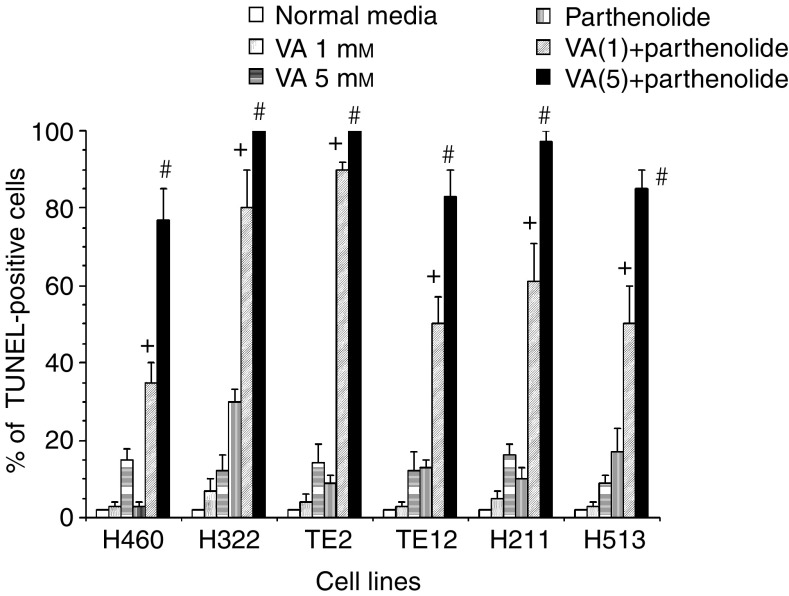
Supra-additive induction of apoptosis following concurrent exposure of cultured thoracic cancer cells to the combinations of VA (1.0 or 5.0 mM) and Parthenolide (30 *μ*M) (^+^*P*<0.05–0.01 combination effects of VA(1.0 mM)+Parthenolide *vs* the sum of individual drug effects and ^#^*P*<0.0001 combination effects of VA(5.0 mM)+Parthenolide *vs* the sum of individual drug effects). Data are expressed as mean±s.e.m. of three independent experiments.

Suppression of VA-mediated NF-*κ*B activation by the PKC inhibitor CC or by the kinase inhibitors STP and UCN-01 was correlated with significant synergistic induction of apoptosis in NSCLC cells H460, EsC cells TE12 or MPM cells H211 at both the clinically relevant VA concentration of 1.0 mM and particularly at higher VA concentration of 5.0 mM (Figure 7A). We further investigated the ability of UCN-01 to mediate synergistic cytotoxic effect in combination with VA in H322, H460, TE2 and H513 (Figure 7B). Whereas <25% of cultured thoracic cancer cells treated with either VA (1.0 or 5.0 mM) or UCN-01 (500 nM) underwent apoptosis, 60–85% of these cells were TUNEL-positive 48 h after the onset of exposure to combinations of VA+UCN-01. The magnitude of the interaction between UCN-01 (at 500 nM) and VA (1.0 or 5.0 mM) to mediate additive (H460 and H513) or supra-additive (H322, TE2, TE12, H211) apoptotic cell death varied between cell lines at VA at 1.0 mM but uniformly profound at higher concentration of VA of 5.0 mM in all cell lines (Figure 7B). At the indicated concentrations, STP (200 nM) was a more rapid and potent inhibitor of PKC than UCN-01 (500 nM) (Figure 2). As PKC was previously demonstrated to be a positive regulator of HDACI-mediated NF-*κ*B activation (Maxhimer *et al*, 2005), it was not surprising to observe that STP was significantly more potent than UCN-01 in abrogating VA-mediated activation of NF-*κ*B (Figure 4). This was positively correlated with the more pronounced induction of apoptosis by the STP+VA (1.0 mM) combination (Figure 8). We next investigated the effect of direct inhibition of NF-*κ*B by using the pharmacologic inhibitor Parthenolide that interferes with NF-*κ*B activation by targeting the I*κ*B kinase complex via inhibition of NIK- and MEKK1-induced activation of IKK*α* and IKK*β* (Murphy *et al*, 1988; Bork *et al*, 1997; Hehner *et al*, 1999; Curry *et al*, 2004). Parthenolide at 30 *μ*M completely abrogated VA-induced NF-*κ*B transcriptional activation in the representative cultured thoracic cancer cells (Figure 4). This was associated with substantial increase of apoptosis of cells treated with VA+Parthenolide, particularly at the low, clinically relevant concentration of VA of 1.0 mM (Figures 7A and 9). These findings were in complete agreement with observations previously reported by our group (Maxhimer *et al*, 2005) as well as by others (Mayo *et al*, 2003, Rundall *et al*, 2004) and confirmed the notion that activation of NF-*κ*B in HDACI-treated cells impeded the ability of HDACI to effectively mediate cell death.

## DISCUSSION

In this study, we attempted to evaluate the possibility of enhancing the cytotoxic effect of VA, a commonly used antiepileptic drug with HDAC-inhibitory activity, on cultured thoracic cancer cells by combining it with the kinase inhibitor STP or its clinically relevant analogue UCN-01. Valproic acid, by itself, is not a very efficient anticancer agent, at least for thoracic cancers. It exerts a mild growth-inhibitory effect in cultured thoracic cancer cells with the *in vitro* IC_50_'s ranging from 4.0 to 8.0 mM. This is mainly attributable to cell cycle arrest at the G1/S checkpoint and very weak induction of apoptosis. Similar to other well-established HDACIs like TSA or SAHA, VA significantly stimulated the NF-*κ*B transcriptional activity in cultured thoracic cancer cells. The kinetics of VA-induced NF-*κ*B activation was somewhat delayed with discernible increase of the transcriptional activity being observed only 18–24 h of drug exposure as compared to the rapid NF-*κ*B activation (6–12 h after the onset of drug exposure) following TSA treatment (data not shown). Partial to complete inhibition of VA-induced activation of NF-*κ*B was achieved by concurrent exposure of VA-treated cells to UCN-01, CC or STP. As HDACI-mediated activation of NF-*κ*B transcriptional activity is positively regulated by PKC, it is conceivable that UCN-01 or STP, being potent PKC inhibitor (Figures 1A and 2), suppressed NF-*κ*B activation in VA-treated cells via this mechanism. As a matter of fact, high levels of p-adducin in VA-treated cells were totally abrogated by either STP or UCN-01 (Figure 2). Suppression of NF-*κ*B activation by these kinase inhibitors was paralleled by the supra-additive induction of apoptosis in combination-treated cells, the magnitude of which appeared to correlate with the degrees of suppressed NF-*κ*B transcriptional activity (for instance, STP *vs* UCN-01). Staurosporine (200 nM) was more efficient than UCN-01 (500 nM) in mediating profound apoptosis of cells concurrently treated with the clinically relevant concentration of VA of 1.0 mM (Figure 8). Inhibition of NF-*κ*B transcriptional activity in VA-treated cells by STP or UCN-01 has functional significance and directly contributed to the synergistic enhancement of apoptosis and not merely a secondary event or a surrogate marker of downregulation of PKC activity. In concordance with previous reports (Rundall *et al*, 2004), including our own (Maxhimer *et al*, 2005), direct inhibition of NF-*κ*B by using Parthenolide profoundly potentiated apoptosis of VA-treated cells. Parthenolide, a sesquiterpene lactone that was first isolated from the feverfew herb (*Tanacetum parthenium*) is a relatively specific small molecule inhibitor of NF-*κ*B (Bork *et al*, 1997; Hehner *et al*, 1999). Parthenolide has undergone a phase I clinical trial as an anticancer agent (Curry *et al*, 2004). Thus, combining clinically applicable HDACIs such as Depsipetide, MS-275 or SAHA with NF-*κ*B inhibitor like Parthenolide to enhance the anticancer effect of HDACIs may have clinically translatable potential. Targeting the NF-*κ*B signalling pathway, similar to targeting PKC, using pharmacologic inhibitors to enhance the cytotoxic effects of VA as well as other HDACIs in cancers may have practical clinical application. This is the current focus of our laboratory investigation.

Staurosporine and UCN-01, in addition to being PKC inhibitors, have also been shown to inhibit the kinase activity of PDK1, leading to the suppression of Akt phosphorylation and activation among other downstream targets (Sato *et al*, 2002). Sato *et al* (2004) have also demonstrated that PDK1 may directly phosphorylate and activate MEK and ERK1/2. It is therefore conceivable that STP or UCN-01 can mediate suppression of Akt and/or ERK1/2 activation. Indeed, UCN-01 has been shown to downregulate Akt activation (but concomitantly stimulate ERK1/2) in head and neck squamous cell carcinoma (Amornphimoltham *et al*, 2004; Kondapaka *et al*, 2004). Continuous exposure of thoracic cancer cells to UCN-01 (250–1000 nM) in 10% FCS RPMI culture medium (in contrast to low serum conditions as were previously described (Amornphimoltham *et al*, 2004; Kondapaka *et al*, 2004)) led to a profound but short-lived reduction of pAkt at 1 h after drug exposure followed by a strong activation of Akt at 24 h time point. On the other hand, there was a profound and durable inhibition of ERK1/2 activation in UCN-01-treated cells. This is in direct contrast to previous studies that described activation of MEK/ERK1/2 by UCN-01 in head/neck squamous cell carcinoma cell lines (Amornphimoltham *et al*, 2004; Kondapaka *et al*, 2004) or leukaemia cell lines (Dai *et al*, 2001, 2002). The mechanism of this discrepancy is not clear and may relate to the intrinsic difference of cell lines and experimental conditions employed. Staurosporine profoundly inhibited ERK1/2 activation and at the same time mediated phosphorylation of Akt in cultured thoracic cancer cells within the similar time interval. This effect of STP on Akt phosphorylation was surprising, given the fact that its closely related analogue UCN-01 suppressed Akt phosphorylation (Sato *et al*, 2002; Amornphimoltham *et al*, 2004; Kondapaka *et al*, 2004; and also our own observation). This was totally unexpected but very reproducible in many independent experiments with our cell lines and the molecular basis of this discrepancy was unclear. Not surprising, however, STP or UCN-01 exerted a potent inhibitory effect on PKC activity indicated by a profound time- and dose-dependent depletion of p-adducin levels in treated H460 or TE12 cells. Grant and co-workers have observed reduction of MEK/ERK1/2 activity in cells treated with combinations of HDACIs and other kinase inhibitors, including perifosine, 17-AAG or LY294002 that mediated substantial apoptosis (Dai *et al*, 2001; Rahmani *et al*, 2003b, 2005). Downregulation of MEK/ERK1/2 plays an important role in the synergistic induction of apoptosis as forced expression of constitutively active MEK abrogates the cytotoxic effects of these drug combinations (Dai *et al*, 2001; Rahmani *et al*, 2003b, 2005). It was further indicated that LY294002 enhances HDACI cytotoxicity via downregulation of MEK/ERK1/2 activity but not via abrogation of Akt activation (Rahmani *et al*, 2003b). It, therefore, appears from the literature that abrogation of MEK/ERK1/2 signalling may have more dominant impact on the enhancement of HDACI cytotoxicity in cancer cells than inhibition of Akt-mediated signalling pathway. UCN-01 and STP consistently suppressed MEK/ERK1/2 and PKC activity in VA-treated cells and correlated well with its synergistic interaction with VA to induce massive apoptosis. It is not known if UCN-01- or STP-mediated inhibition of VA-induced NF-*κ*B activation, while conceivably attributable to their PKC-inhibitory effect, can be mechanistically linked to their ability to downregulate ERK1/2 or Akt activity. Inhibition of MEK/ERK1/2 and PKC by STP or UCN-01 can both contribute to their VA sensitisation effect in thoracic cancer cells, yet the relative contribution of each to this effect is not clear at present. Even though we did not focus our investigation on the effect of UCN-01 on PDK1 kinase activity, it is conceivable that via inhibition of PDK1, which occupies an apical position in regulating multiple downstream kinases, UCN-01 can simultaneously inhibit multiple parallel targets including PKC, MEK/ERK1/2 and Akt.

In summary, UCN-01 and VA, at clinically achievable drug concentrations and conditions (for instance 1.0 mM of VA and 500 nM of UCN-01) (Brodie and Dichter, 1996; Sausville *et al*, 2001), interact to mediate additive and supra-additive induction of apoptosis of cultured thoracic cancer cells. This chemosensitisation effect of UCN-0-1 in VA-treated cells is most likely secondary to its effect on downregulation of essential signal transduction pathways (PKC, ERK1/2, NF-*κ*B) that regulate HDACI-induced apoptosis. Moreover, our study further confirms NF-*κ*B as a valuable target in the ongoing development of clinically applicable strategies to potentiate the anticancer effect of HDACIs. Within this context, direct targeting of NF-*κ*B using clinically relevant pharmacologic inhibitor like Parthenolide appears to be more effective in enhancing the anticancer property of VA, particularly at concentration such as 1.0 mM that can be readily achievable in the clinic.
